# Specialized membrane domains of plasmodesmata, plant intercellular nanopores

**DOI:** 10.3389/fpls.2014.00507

**Published:** 2014-09-30

**Authors:** Emmanuelle M. Bayer, Sébastien Mongrand, Jens Tilsner

**Affiliations:** ^1^Laboratory of Membrane Biogenesis, University of BordeauxBordeaux, France; ^2^Biomedical Sciences Research Complex, University of St AndrewsFife, UK; ^3^Cell and Molecular Sciences, The James Hutton InstituteDundee, UK

**Keywords:** plasmodesmata, lipid rafts, membrane microdomains, membrane curvature, plasma membrane, endoplasmic reticulum, super-resolution microscopy, protein-lipid interaction

Plasmodesmata (PD) are plant-specific membrane-lined channels that connect neighboring cells across the cell wall and are indispensable for intercellular communication, development and defense against pathogens. They consist of concentric membrane tubules of the plasma membrane (PM) on the outside and endoplasmic reticulum (ER) on the inside. The biophysical properties and molecular composition of both membranes are most likely distinct from the respective bulk membranes with which they are continuous. This specialization of PD membranes is expected to guarantee not only the compartmentalization of PD-related function but also to accommodate the requirement for highly curved membrane organization (Mongrand et al., 2010; Tilsner et al., 2011).

This Research Topic brings together researchers from a variety of areas to apply the significant recent advances in understanding how the interactions of lipids and proteins influence the behavior and spatial/functional compartmentalization of biological membranes on PD-related questions.

The first several contributions are focussed on the molecular and physical properties of the PD plasma membrane (PD-PM). The PD-PM contains a different set of proteins than the PM outside the channels and does not permit free diffusion of membrane components between cells, indicating that it is laterally segregated from the bulk PM and forms a membrane microdomain (or several). In line with a view of microdomains as signaling “hubs,” PD have recently been emerging as important sites of pathogen-related and developmental signaling. Faulkner (2013) reviews the currently identified PD-located receptors and suggests that sub-division of the PD-PM into microdomains, be it raft-like or tetraspanin webs, may facilitate signaling processes through the local clustering of membrane components. Preferential compartmentation of proteins but also lipids into membrane microdomains have been postulated for many cell types, but have long been difficult to directly visualize *in vivo*. Owen and Gaus (2013) and Truong-Quang and Lenne (2014) both review how recent advantages in light microscopy that allow imaging below the diffraction limit can be used to obtain new insights into the dynamics of microdomains and to draw conclusions on the mechanism of their formation. Truong-Quang and Lenne review internal structuring as well as higher-order clustering of microdomains. Owen and Gaus discuss their recent findings from direct imaging of PM lipid order *in vivo*. They found the PM to consist of ~75% liquid-ordered (L_o_) and 25% liquid-disordered (L_d_) sub-resolution microdomains and postulate that small changes in lipid phase distribution can induce rapid large-scale changes in protein geometry of the PM when a lipid phase switches from being the “island” to the “percolating” phase and vice versa.

So far no data exist as to how lateral membrane heterogeneity and compartmentalization of biological processes are achieved at PD. In other words, how are locally confined PD membrane sites established and maintained within the pore, despite their continuity with the bulk membranes outside PD? What mechanisms restrict lateral mobility of proteins and possibly lipids along the PD membranes?

A number of articles ask how a laterally segregated PM domain could be maintained at PD (and elsewhere). One potential mechanism for microdomain formation is the “picket fence” model which suggests that, in mammalian cells at least, PM domains are corralled by structural elements attached to the membrane and underlying cytoskeleton. In plant cells different mechanisms might be at work. Martinière and Runions (2013) review their recent experimental findings showing that compared to animal cells, most of the plant PM-resident proteins display a low mobility and that restricted lateral diffusion depends mostly on the cell wall. Intricate connections between the PD-PM and surrounding wall have been observed and are likely to contribute to the specialization of this membrane domain.

Boutté and Moreau (2014) review the role of small GTPases in PM partitioning and suggest that such mechanisms could also act at PD. Several small GTPases have been found in the PD proteome and could potentially be involved in specifying the PD-PM.

In line with the idea that the PD-PM may cluster L_o_ sterol and sphingolipid enriched raft microdomains, a number of articles provide insights about the potential contribution of lipid phase separation to the selective lateral segregation of PD components. de Almeida and Joly (2014) suggest that nano-scale lipid phase separation may also include the formation of solid-ordered/gel (S_o_) phases around nucleating oligomers of membrane-integral proteins or lipids, which could stabilize membrane microdomains for longer time spans than the L_o_ domains of the conventional raft hypothesis. Whilst still speculative at this stage, such a model could potentially provide an explanation for the restricted lateral diffusion within the PD-PM. On their side Bagatolli and Mouritsen (2013) ask whether the now-classical fluid mosaic model, together with the raft hypothesis based on segregation of L_o_ and L_d_ phases is really suitable to describe the lateral segregation that has been observed in biological and model membranes. They conclude that the molecular shape of membrane lipids, which introduces curvature stress into bilayers, needs to be taken into consideration when investigating membrane lateral segregation, and the distribution and activity of proteins in the bilayer.

Although the involvement of lipids in PD functionality has been suggested, we actually know very little about PD lipid constituents. As a way to determine whether sterol- and sphingolipid-enriched microdomains contribute to PD membranes as suggested by the presence of raft and tetraspanin protein markers, Naulin et al. (2014) propose an original approach based on Mass Spectrometry (MS) and Atomic Force Microscopy (AFM) directly on purified PD membranes. Whereas AFM could be applied to identify microdomains based on topological parameters, MS approaches could resolve the PD lipid profile and identify intact membrane proteins and the stoichiometry and nature of lipids bound to them.

In animal cells, the recently discovered tunneling nanotubes (TNTs) also connect the PM between cells via a highly curved membrane tubule, although they differ from PD by the lack of an ER connection. Delage and Zurzolo (2013) review the current knowledge of the lipid composition of TNTs and suggest that comparisons with PD lipids will be informative in understanding the functional similarities and differences between these structures. They suggest that lipids may play a critical role in the formation and stability of these highly curved structures.

Because sphingolipids are typically enriched in raft-like microdomains, González-Solís et al. (2014) describe Arabidopsis mutants defective in specific steps of sphingolipid synthesis which could be used to dissect the contribution of sphingolipids to putative PD-PM microdomains. Sphingoid bases, breakdown products of sphingolipids, also act as second messengers in pathogen-induced programmed cell death and the authors suggest that like other defense-related signaling this pathway could act at PD.

Linking lipids and the regulation of PD permeability, De Storme and Geelen (2014) discuss the potential involvement of sterols in the regulation of PD aperture through the deposition and removal of the water-insoluble cell wall polysaccharide callose (β-1,3-glucan) at the neck region of the pore, which leads to a physical constriction of the PD opening. Callose synthases are transmembrane complexes like cellulose synthases, and callose-degrading β-1,3-glucanases are membrane-anchored apoplastic enzymes. The site-specific turnover of callose at PD therefore also requires targeting of proteins and membranes to the specific domain at PD. De Storme and Geelen provide an overview of callose metabolism at PD and note similarities in the developmental phenotypes of mutations in callose synthases with those of sterol synthesis mutants. They speculate that structural sterols may play a role in PD callose turnover, either as constituents of the PD-PM, or as substrates for callose synthase.

Through phylogenetic analyses, Gaudioso-Pedraza and Benitez-Alfonso (2014) identify a subgroup of the Glycosyl hydrolases family 17 (GHL17), called clade alpha, that have diverged during plant land colonization and would therefore correlate with the appearance of complex PD. Hence, all the callose degrading enzymes identified so far belong to this specific clade. They suggest that a portion of the alpha clade GHL17 membrane proteins have evolved in embryophytes differently from other clades to specifically target PD.

Taking advantage of the growing understanding of callose turnover at PD, Yadav et al. (2014) present their *icals3m* system, which enables inducible overaccumulation of callose at PD and a concomitant reduction in intercellular communication, to study the role of PD during development. Given the intricate links between membrane trafficking to the PD-PM and callose metabolism at the pores, this system could perhaps also be combined with lipid biosynthesis mutants to investigate the targeting mechanism of PD-specific callose synthesis.

One possible way for delivery of β-1,3-glucanases to PD is suggested by Paul et al. (2014). These authors have found that lipid bodies, ER-derived structures surrounded by a lipid monolayer, are positioned at PD openings in meristematic cells prior to the removal of callose plugs at the onset of dormancy release. These lipid bodies carry β-1,3-glucanases which are probably delivered to PD bypassing the conventional secretory pathway, possibly by interacting with cytofacial plasma membrane rafts. Paul et al. speculate that a similar mechanism might also function in the delivery of other PD components.

PD are dynamically modified during plant development, and Demchenko et al. (2014) present their research on specialized cells hosting actinorhizal, nitrogen-fixing bacteria in *Casuarina* nodules, where PD become very narrow and embedded in a lignified cell wall. It appears that this modification is connected to a loss of some of the specialized membrane features of PD as the desmotubule may be removed and the wall surrounding the PD-PM is devoid of callose. Whether these modifications represent stages of PD degradation and permanent closure, or to the contrary the transformation of PD into unregulated open channels, remains to be seen.

Shifting the focus from the PD-PM to the PD-ER, the desmotubule needs to be connected to the cortical ER at either side. If this connection severs, or when new secondary PD are laid down in existing walls, the PD-ER needs to be (re)connected to the cortical ER outside of the channel. A family of proteins called atlastins was recently identified which mediates this type of homotypic ER tubule fusion. Zhang and Hu (2013) review the current knowledge about plant atlastins, also known as RHD3s.

The ability to connect the ER across the cell wall may be a precondition for the ability to form secondary PD across existing cell walls. Evkaikina et al. (2014) review the evolutionary origins of secondary PD formation and its link to the organization of the shoot apical meristem, which uses transport of miRNAs and transcription factors through PD to establish polarity axes. It is likely that the ability to form new PD involved evolution of both the cell's membrane organizing and cell wall modifying machineries.

The desmotubular ER is extremely constricted, to about the dimensions of a vesicle fission stalk, and in EM images sometimes seems to contain an electron-dense “central rod” that may correspond to lipid headgroups in a virtually lumen-less tubule. Certain types of lipids could favor such an arrangement. Jouhet (2013) reviews the curvature-related biophysical properties of membrane lipids and highlights the possibility that the desmotubular ER may assume an unusual, non-bilayer lipid phase.

We hope that the articles collected in this Research Topic/e-book will stimulate discussion and new experimental approaches in plasmodesmata research.

## Conflict of interest statement

The authors declare that the research was conducted in the absence of any commercial or financial relationships that could be construed as a potential conflict of interest.
